# Using Artificial Intelligence to Enhance Evidence Search and Synthesis

**DOI:** 10.1097/AJN.0000000000000288

**Published:** 2026-04-23

**Authors:** Susan Bell, Michael Ackerman, Jacalyn Buck, Andrew Frueh, Jillian Maitland, Kathy Piotrowski, Maria Sokol, Todd E. Tussing, Cindy Zellefrow, Molly McNett

**Affiliations:** **Susan Bell** is program director at the Helene Fuld Health Trust National Institute for Evidence-Based Practice in Nursing and Healthcare at The Ohio State University (OSU) College of Nursing, Columbus, OH, where **Michael Ackerman** is a clinical professor and director of the Master of Healthcare Innovation Program and the Center for Healthcare Innovation and Leadership, **Jacalyn Buck** is a clinical professor and executive director for academic partnerships, **Andrew Frueh** is a project manager, **Kathy Piotrowski** and **Todd E. Tussing** are associate clinical professors, **Cindy Zellefrow** is an assistant professor and DNP project coordinator, and **Molly McNett** is associate dean for evidence-based practice and implementation science and the Helene Fuld Endowed Professor of Evidence-Based Practice. **Jillian Maitland** is associate chief nursing officer for women and infants and emergency services at the OSU Wexner Medical Center, Columbus, OH. **Maria Sokol** is a clinical abstraction specialist, Cleveland Clinic, Cleveland, OH. Contact author: Susan Bell, bell.15@osu.edu. The authors have disclosed no potential conflicts of interest, financial or otherwise.

## Abstract

Accelerating the path from research to practice

Integrating evidence into practice is essential for shared decision-making and equitable, patient-centered care. However, identifying and synthesizing the best available research to inform clinical decisions is time-consuming and complex. Effective literature searching and synthesis typically requires multiple, methodical steps. In some cases, established clinical practice guidelines developed by expert panels exist, expediting the translation of evidence into practice while maintaining quality and accuracy. Yet not all areas of clinical practices have established guidelines; when this is the case, clinicians must search and synthesize research evidence to facilitate evidence-based practice (EBP) when making decisions about patient care.

The process of EBP relies on using the best available evidence to inform clinical decision-making. The initial steps of EBP include structured, systematic approaches for literature searching, critical appraisal, and evidence synthesis to yield reliable and clinically useful information. However, if these processes are applied inconsistently or without methodological rigor, resulting conclusions may be biased or unreliable.

Despite its recognized importance, these initial steps of EBP are fraught with obstacles. Conducting comprehensive literature reviews and synthesizing data manually is resource-intensive. Even experienced clinicians may find the process burdensome, requiring several months to identify and implement best practices. Challenges are further exacerbated by competing priorities, inadequate funding, limited access to necessary resources and informatics support, and insufficient stakeholder engagement. The rapid advancement of artificial intelligence (AI) capabilities presents new opportunities to streamline and enhance the EBP process. The purpose of this article is to present findings from an EBP–AI task force that evaluated AI tools for searching, synthesis, and appraisal in order to outline key considerations and preliminary recommendations for their responsible use in the EBP process.

## INTEGRATING AI INTO EBP

Integrating AI tools into evidence-based decision-making holds potential to enhance efficiency and support timely, informed clinical care.^1^ AI can rapidly process large volumes of data, aiding in evidence identification, evaluation, and application for health care decision-making. However, successful integration requires thoughtful strategies that foster provider trust, usability, and workflow alignment.

To address practical challenges, AI technologies, including AI tools that generate text (large language models [LLMs]), chatbots, and virtual assistants, offer tools supporting clinicians throughout the evidence to practice process. In practice settings, clinicians can use AI tools in everyday hospital projects. For example, AI can be used to identify and summarize best practice for quality improvement efforts such as reducing pressure injuries or catheter-associated infections. In each project, although AI can be integrated into daily work to save time and support evidence-based decisions, it should complement the EBP process without replacing professional judgment. The following sections present the usage of AI in the EBP process, offering best practice considerations for generating clinical questions, literature searching, evaluation, and synthesis.

**Formulating the clinical question**. Searching for evidence begins with developing a well-structured, searchable question. Well-developed questions improve search efficiency by reducing time and increasing the relevance and volume of retrieved evidence. AI tools assist by suggesting relevant keywords, subject headings, synonyms, and Boolean operators; however, the quality of output depends on user prompting and the capabilities and version of the AI model.^2^ EBP mentors can use AI tools to format PICOT (population, intervention, comparison, outcome, time) questions; generate search terms; and develop evidence summaries.

The literature and task force findings on AI-assisted clinical query formulation are mixed. Research questions are typically specific and narrowly focused, while EBP questions often benefit from broader wording. Broad questions allow for a global view of literature, helping identify terminology, interventions, and approaches that may not have been previously considered. While AI tools show potential for enhancing development of clinical questions and supporting literature retrieval, accuracy and contextual relevance remain variable. AI tools may identify relevant keywords and provide initial drafts of a clinical question.^2^ However, our team and others found human oversight is still necessary to ensure accuracy, appropriateness, and alignment with the goals of the clinical issue.

**Searching the literature**. The next step in the EBP process is conducting a comprehensive literature search. The exponential growth of evidence has made reliance on manual search methods increasingly impractical. Consistent with previous findings from Orgeolet and colleagues, our task force found faster retrieval with AI-assisted searching compared to traditional methods and the capability to generate search strings across multiple databases.^3^ In EBP, literature searching is an iterative process, which often leads to refining or creating new clinical questions to address different facets of a query.^4^,^5^ As such, reliance on a single search using AI tools should be avoided, as our team found improved accuracy with multiple, iterative searching.

Despite potential benefits, our task force found AI tools may have access and function limitations. Access barriers include the inability of some AI tools to search subscription-based databases such as CINAHL, Scopus, and Embase, where much peer-reviewed literature resides, thus resulting in important gaps. Function issues found by our team and others include vague references; incorrect journal information; fabricated citations; and failure to use synonyms, plurals, alternate spellings, or truncation to expand searches.^2^,^6^,^7^ Several academic search platforms such as PubMed and EBSCO have incorporated AI-enhanced search features to reduce these problems. Improved model training and advances in AI technology have additionally reduced these issues but not eliminated them entirely. While AI-generated searches may yield more results, their inconsistency and lack of reliability underscore the need for continued expert led database searching and source verification.

However, AI tools may be used to augment human-directed literature searching. Various applications are available to suggest search strings, MeSH (medical subject headings) terms, and databases to guide the literature search. When using an AI tool to search, to improve the quality and accuracy of the search, it is important to verify which sources and databases are being used. Verification of MeSH terms is also required, as our team found some AI tools generated false terms that did not exist. Our team found that effective, iterative prompting is an important skill that increases the likelihood of valid and applicable search results. Prompting should start with broad questions, refining these iteratively, asking follow-up questions when responses are too general, and using multiple prompts. Table 1 displays examples of effective prompts.

**Table 1.** Examples of Effective AI Prompting

Best Practice	Good Example	Poor Example	Rationale
Clinical query	What is the evidence for using metformin as treatment for type 2 diabetes in adults over 65 with chronic kidney disease?	What's the best diabetes medication?	Specific questions yield more clinically relevant information.
Evidence quality	Summarize the evidence for cognitive behavioral therapy for insomnia and rate the quality of evidence using the GRADE approach.	Can cognitive behavioral therapy help insomnia?	Asking for a quality assessment improves the strength of the evidence.
Evidence hierarchy	Provide information on postoperative pain management with acetaminophen, prioritizing systematic reviews from 2020 to 2025.	What is the benefit of using acetaminophen for pain after surgery?	Prioritizing higher-quality evidence ensures more reliable information.
Methodology	Explain how you're evaluating the strength of the evidence when discussing hormone replacement therapy for menopausal symptoms.	What are the pros of using hormone replacement therapy?	Requesting transparency about assessment methods increases understanding about how conclusions are reached.
Implementation	Provide evidence-based strategies for implementing a fall prevention program in a long-term care facility.	How do I prevent falls?	Asking for evidence-based strategies bridges the clinical practice gap.
Patient values	How should patient preferences be integrated when discussing evidence-based guidelines for anticoagulation in a 50-year-old woman with deep vein thrombosis?	How should apixaban be prescribed?	Incorporating patient values aligns with shared decision-making.
Knowledge gap	What are the knowledge gaps in nonpharmacological management of hypertension in African American men?	What are nonpharmacological options to treat hypertension?	Identifying knowledge gaps highlights areas needing clinical judgment.
Evidence synthesis	Synthesize the evidence comparing β-blockers and calcium channel blockers for essential hypertension management in adults over 65.	Are β-blockers still being recommended?	Requesting synthesis encourages integration of multiple studies.
Context	How should evidence-based pressure injury prevention guidelines from the Association of periOperative Registered Nurses be implemented in the operating room?	How do you prevent pressure injuries?	Integrating professional guidelines provides context for real-world application.

**Critical appraisal, evaluation, and synthesis**. Beyond literature searching, AI has potential in critical appraisal, evaluation, and synthesis of evidence. Tools such as PaperDigest, ChatPDF, and OpenAI's ChatGPT can summarize text, answer targeted questions, and extract data from full-text articles. Some platforms automate synthesis tables and concept maps, streamlining the organization of findings. However, the user should upload preselected articles to use this function and not rely solely on these programs to search and synthesize the evidence, as sources for searching may not include peer-reviewed research. Uploaded documents should include public domain or open access content unless publisher or institutional permissions have been obtained.

AI appraisal tools can assess methodological features such as sample size, randomization, and potential bias, filtering low-reliability studies to reduce manual work.^8^ Grouping and visualization techniques can reveal relationships and identify themes. LLMs can summarize complex findings, uncover patterns, and generate insights that may be overlooked, saving time.

While AI may address time constraints, concerns remain regarding the quality and trustworthiness of AI-generated syntheses. Issues of validity, reliability, replicability, and bias underscore risks of overreliance.^9^ It has been demonstrated that current LLMs alone perform worse than human reviewers in appraisal^10^; thus fact-checking, accountability, and human verification remain critical. Table 2,^9^,^11^-^14^ lists important considerations and recommended actions to evaluate the validity of AI outputs.

**Table 2.** Evaluating AI Output Validity

Criterion	Questions to Ask	Red Flags
State of the art	Is the information based on up-to-date evidence? Does it acknowledge AI's cutoff date?^11^	References to outdated guidelines^12^ No mention of recent developments Failure to recognize knowledge limitations
Evidence quality	Does the response select high-quality evidence?	Overreliance on observational studies Equally weights a range of cited sources
Completeness	Does the response address all aspects of your query? Are important perspectives or alternatives omitted?	One-sided presentation of evidence Misses key treatment options Overlooks important patient populations
Practice standards	Does the information align with current clinical practice guidelines from reputable organizations?	Recommendations that contradict major guidelines without explanation Failure to mention guideline disagreements
References	Are specific studies or guidelines cited? Can the claims be verified against primary sources?^9^	Vague references (“studies show”) Citation of nonexistent papers Misrepresentation of study findings
Uncertainty	Does the response acknowledge areas of uncertainty in the evidence?	Overconfident assertions in areas with limited evidence False certainty in controversial areas
Statistical accuracy	Are statistics, numbers, and effect sizes accurately reported and interpreted?^13^	Misinterpretation of P values
Clinical relevance	Does the response connect evidence to practical clinical applications and patient-specific factors?^14^	Theoretical discussion without practical application Failure to consider patient context^14^
Bias	Does the response acknowledge potential biases in the evidence?	Evidence supports only one perspective Failure to note industry funding
Logic	Is the reasoning process logical? Do conclusions follow from premises?	Logic errors Clear contradictions in the output response

**Misinformation and transparency risks**. Like many others, Jeyaraman and colleagues found that LLMs frequently generate factually incorrect or fabricated content, an AI output termed “hallucination.”^15^ Such content often appears coherent and authoritative, creating substantial risks for clinicians. In health care, where decisions must rest on rigorously vetted evidence, such inaccuracies can cause serious adverse outcomes.

AI-generated content requires systematic manual review by qualified professionals to ensure alignment with peer-reviewed literature, clinical guidelines, and institutional protocols. Without robust human oversight, embedding misinformation in clinical workflows remains an unacceptably high risk.

Transparency of searching and synthesis of the literature remains difficult in AI programs. Deep learning AI systems often function as “black boxes” (unclear decision-making steps) with minimal insight into how conclusive statements were made. When AI tools are asked to explain reasoning, their outputs may be prone to hallucination, thus compounding the inaccuracy of original responses.^15^ Despite technical advances to address some of these limitations, human judgment remains indispensable.

In health care, AI may assist in literature reviews, guideline summarization, and decision support. Final judgments, however, must remain with trained professionals who interpret outputs within the broader context of patient needs, ethics, and institutional standards. This reflects an early vision of AI focused on supporting human intelligence rather than replacing it, reinforcing the importance of integrating AI with human expertise.

**Costs and return on investment**. Our team found cost is a critical factor when selecting AI tools for evidence-based decision-making. Many platforms, such as Claude, ChatGPT, Consensus, Elicit, NotebookLM, and Perplexity offer free versions with limited functionality, while advanced features require paid subscriptions. The pricing, availability, and features of AI tools change frequently and therefore require ongoing checking. For those beginning to use AI, it is reasonable to start with general-purpose tools such as ChatGPT that support basic EBP queries. Users, however, should set limits when searching or use prompts that target only peer-reviewed research. Clinicians can use free tools for preliminary queries and then verify findings with institutional resources, while reserving subscription-based services for complex inquiries or synthesis.

Beyond direct costs involved in using tools requiring subscription, AI tools can improve efficiency and save clinicians' time by accelerating evidence retrieval and synthesis. However, achieving positive return on investment (ROI) requires accounting for training and onboarding costs. Institutions must invest in upskilling clinicians, adapting workflows, and ensuring user proficiency for safe AI use.

When evaluating ROI, health care organizations must consider not only pricing but also infrastructure and cultural readiness. Legacy informatics systems, lack of interoperability, and resistance to change can impede implementation and reduce realized value. To maximize ROI, organizations should adopt comprehensive strategies including infrastructure modernization, ethical and regulatory safeguards, and active stakeholder involvement.

**Ethical and legal considerations**. AI offers potential in implementing evidence into health care yet raises complex ethical and legal questions. Addressing challenges requires interdisciplinary collaboration among clinicians, ethicists, legal scholars, and regulators. Zhang and Zhang advocate for AI ethics committees at governmental and institutional levels.^13^ These bodies could monitor privacy, bias, explainability, and accountability. Furthermore, malpractice and liability laws are poorly equipped to address AI's role in clinical decisions,^16^ leaving both patients and providers in uncertain territory.

Ethical concerns surround transparency, accountability, bias, and data quality.^2^,^15^ Floridi and colleagues describe this as a “dual advantage” of ethics: oversight not only helps organizations pursue socially acceptable innovations but also mitigates potential harms before they occur.^17^ The risk of overreliance on AI may erode clinicians' critical thinking and independent decision-making abilities. Clinicians must understand the implications of AI-driven decisions, particularly regarding responsibility for errors. Biases embedded in AI models, whether from poorly formulated questions or incomplete synthesis, may lead to skewed or unsafe recommendations.^2^ Liability remains unresolved when harm occurs. Does responsibility lie with the developer, provider, or institution? This is compounded by the lack of transparency of many AI systems, which obscures how decisions are generated.

Given these complexities, AI must be integrated into health care with caution and transparency. Combining human expertise with AI outputs is essential to ensure safe, reliable, and patient-centered care. Ethical integration is not optional but a prerequisite for sustainable, trustworthy use of AI in evidence-based health care.

## BEST PRACTICE CONSIDERATIONS AND PRELIMINARY RECOMMENDATIONS

Ethical and legal considerations highlight the risks of adopting AI in health care. Equally important are the practical strategies needed for safe, effective use. Beyond regulation, clinicians and organizations must integrate AI into evidence-based workflows in ways that foster trust, usability, and efficiency. Table 3 highlights 13 best practice considerations and preliminary recommendations from the task force for use of AI in the evidence translation process. Best practice considerations focus on optimizing existing AI tools to augment literature search and synthesis. Recommendations emphasize responsible use, transparency in reporting, and methods to verify validity of findings.

**Table 3.** Best Practice Considerations and 13 Preliminary Recommendations

Stage of Evidence Translation	Best Practice Considerations	Preliminary Recommendations
Formulating the clinical PICOT question	AI tools can be used to suggest elements of the clinical practice question. identify relevant keywords, subject headings, synonyms, and Boolean operators.	Use AI tools in conjunction with human oversight to ensure precision and accuracy.
Literature searching	AI tools can be used to identify search strings and MeSH terms. find appropriate databases to search. enhance and improve the search. automate the screening process and prioritize articles.	Review references and verify information. Use AI as an adjunct tool. Explore and test various AI tools. Be aware of AI tool limitations and data sources. Use iterative prompting strategies to improve precision of searching.
Critical appraisal, evaluation, and synthesis	AI tools can be used to extract relevant information. summarize study findings. highlight key themes, uncover gaps, and generate insights. generate informative reports.	Be aware of the date of last update (data cutoffs). Human interpretation, critical analysis, and verification from the source documents are critical.
Clinical decision-making and implementation	AI tools can be used to streamline efficiency with research processing. provide updates on current practice. act as an adjunct artificial clinical expert in addition to human decision-making.	Be transparent about how the literature was searched and used to inform clinical decision-making. Cross-check evidence-based recommendations with authoritative sources and/or existing practice guidelines. Be aware of and acknowledge bias potential. Verify that evidence-based recommendations align with internal policies/procedures. Consider the ethical and legal implications.

Improving AI output performance also requires strategies that support provider trust and workflow. AI tools can process large volumes of clinical data to facilitate evidence identification and application, potentially saving time. However, providers must recognize that AI systems have training data limits (date of last major update) and may lack the most recent research, particularly in specialized fields. Clinicians should request study references, verify information independently, and review documentation of evidence-based reasoning. Critical decisions, including treatment and dosing recommendations, must always be cross-checked with authoritative sources. Across health care, a consistent theme remains: AI should complement, not replace, the reflective, patient-centered nature of EBP.

## CONCLUSION

AI has the potential to enhance each stage of evidence implementation in health care. While AI can improve efficiency and consistency by processing large volumes of data and uncovering patterns, its impact on accuracy remains mixed. If used in isolation without human validation, current AI tools can produce inaccurate, unreliable, and fabricated information. These tools also lack transparency in decision-making processes and may perpetuate existing biases.

Despite these limitations, AI presents a promising adjunct to the implementation of evidence-based decision-making. However, its benefits can only be fully realized when paired with human expertise, oversight, and critical evaluation. AI outputs must always be interpreted through the lens of clinical judgment and applied within the context of patient-centered care. Responsible implementation, supported by institutional oversight and continuous evaluation, is essential to mitigate risks and uphold research and clinical integrity. When used thoughtfully and ethically, AI can support, not replace, human reasoning, strengthening clinical practice and improving patient outcomes.
